# Identification of human placenta-derived circular RNAs and autophagy related circRNA-miRNA-mRNA regulatory network in gestational diabetes mellitus

**DOI:** 10.3389/fgene.2022.1050906

**Published:** 2022-11-30

**Authors:** Yindi Bao, Jun Zhang, Yi Liu, Lianzhi Wu, Jing Yang

**Affiliations:** ^1^ Department of Obstetrics and Gynecology, Renmin Hospital of Wuhan University, Wuhan, China; ^2^ Department of Obstetrics and Gynecology, Xiaogan Central Hospital Affiliated to Wuhan University of Science and Technology, Xiaogan, China; ^3^ Reproductive Medical Center/Hubei Medical Clinical Research Center for Assisted Reproductive Technology and Embryonic Development, Renmin Hospital of Wuhan University, Wuhan, China

**Keywords:** circular RNA (circRNA), gestational diabetes mellitus (GDM), autophagy, placenta, ULK1

## Abstract

Gestational diabetes mellitus (GDM) is a metabolic and reproductive disease with serious risks and adverse health effects. However, the pathophysiological mechanism of GDM, especially the roles of circRNAs in its pathogenesis, is largely unknown. The objective of this study was to identify and investigate the roles of circRNAs in GDM. In the current study, placental circRNA expression profiles of normal controls and GDM patients were analyzed using high-throughput sequencing. Bioinformatics analysis identified a total of 4,955 circRNAs, of which 37 circRNAs were significantly deregulated in GDM placentas compared with NC placentas. GO and KEGG enrichment analyses demonstrated that metabolic process-associated terms and metabolic pathways that may be related to GDM were significantly enriched. The biological characteristics of placenta-derived circRNAs, such as their stability and RNase R resistance, were also validated Bioinformatics prediction. Moreover, we constructed the autophagy related circRNA-miRNA-mRNA regulatory network and further functional analysis revealed that the circCDH2–miR-33b-3p–ULK1 axis may be associated with autophagy in the placentas of GDM patients. Our study indicates that aberrant expression of circRNAs may play roles in autophagy in GDM placentas, providing new insights into GDM.

## Introduction

Gestational diabetes mellitus (GDM), which is characterized by transient diabetes caused by insulin resistance and pancreatic *β* cell dysfunction during pregnancy, is a metabolic disease that affects approximately 9%–25% of pregnant women (American Diabetes, 2018; Alejandro et al., 2020; Goyal et al., 2020). GDM accounts for more than 90% of pregnancy diabetes mellitus cases and accounts for 20%–50% of type 2 diabetes mellitus (T2DM) cases that develop postpartum (He and Wu, 2021). GDM-induced dysregulation of blood glucose during pregnancy is associated with a significantly increased risk of perinatal outcomes and with serious short-term and long-term health effects on both mothers and children, such as obesity, preeclampsia, preterm delivery, excessive fetal development, fetal hyperinsulinemia, offspring neurocognitive development and cardiometabolic defects (Chu and Godfrey, 2020; Choudhury and Devi Rajeswari, 2021; Moon and Jang, 2022).

Pregnancy is an important period for both pregnant women and their unborn infants’ long-term health due to possible risk and damage during this sensitive period (Nawabi et al., 2021). During normal pregnancy, pregnant women undergo a transient and physiological decline in insulin sensitivity to ensure that the fetus preferentially receives enough glucose for healthy growth and development. To maintain glucose homeostasis after insulin resistance, β-cells counteract insulin resistance and maintain normal blood glucose by proliferating and synthesizing more insulin (ACOG Practice Bulletin No. 190, 2018; Parsons et al., 1992). However, the pancreas in some women is affected by GDM risk factors. Due to the enhanced insulin resistance, especially in the second and third trimesters of pregnancy, the compensatory activity and proliferation of cells are insufficient (Katra et al., 2016; Moyce and Dolinsky, 2018). When maternal β-cells cannot adapt to the metabolic changes accompanying pregnancy, GDM develops as a result of these changes (Plows et al., 2018). As the placenta is an immunoendocrine organ and due to the roles of placenta-secreted hormones, adipokines and cytokines in the development of insulin resistance, aberrant placental function is considered to be the main cause of GDM (Olmos-Ortiz et al., 2021). Furthermore, hyperglycemia and severe insulin resistance can lead to changes in placental structure and function, such as villous vascularization and enhanced oxidative stress that adversely affect fetal growth and health (Gauster et al., 2012; Carrasco-Wong et al., 2020). Although research on the etiology and pathophysiology of GDM has gradually deepened in recent years, the understanding of the whole pathophysiological mechanism of GDM is still very limited.

Autophagy is a lysosomal-mediated process in eukaryotes that maintains intracellular homeostasis and cell self-renewal under environmental stress. However, excessive autophagy can lead to programmed cell death (Klionsky and Emr, 2000; Mizushima and Komatsu, 2011). Autophagy plays an important role in removing unnecessary proteins and in the degradation pathway of dysfunctional organelles in cells (Filomeni et al., 2015). It also plays an important role in normal placental development, and it is essential for the regulation of trophoblast functions, including invasion and vascular remodeling in extravillous trophoblasts (EVTs) (Nakashima et al., 2019). However, studies on the role of autophagy in GDM have shown inconsistent results. Martino et al. (2016) reported that the expression of AMPK was reduced, whereas that of mTOR and p70S6KB1 was increased, in placentas from obese women with GDM, suggesting that autophagy is inhibited in GDM placentas. Deregulated expression of Beclin-1 and p62 has been observed in GDM placentas, indicating accumulation possibly related to autophagy inhibition (Avagliano et al., 2017). Studies on gestational diabetes in large-for-gestational-age fetuses have shown decreases in placental apoptosis and autophagy (Hung et al., 2020). In contrast, Ji et al. (2017) reported that autophagy was significantly enhanced in GDM placentas and EVTs cultured with high glucose (HG) and that ATG5 plays a critical role in the pathogenesis of GDM. Studies have also found that increased expression of DAPK3, SIRT3 and ULK1 and low expression of miR-193b play important roles in the activation of placental autophagy in GDM patients (Wang et al., 2020a; Han et al., 2020; Ji et al., 2020; Diceglie et al., 2021). Nevertheless, the relationship between autophagy and GDM, as well as the underlying mechanisms, have not been fully elucidated.

Circular RNAs (circRNAs) are a type of noncoding RNA (ncRNA) with closed-loop structures (Chen, 2016). CircRNAs participate in the occurrence and development of many diseases through regulatory functions related to microRNA (miRNA) sponging, transcription and alternative splicing (Chen, 2016; Han et al., 2018). Increasing evidence shows that numerous ncRNAs are dysregulated in the placentas of GDM patients and are related to abnormalities in placental structure, metabolism and function. Despite the identification of multiple miRNAs and long ncRNAs (lncRNAs) and their pathophysiological mechanisms in GDM placentas, few studies have explored the relationship between placental circRNAs and GDM (Du et al., 2021). Recently, four independent studies have reported the expression profiles of circRNAs in GDM placentas. However, perhaps due to the different analysis methods, such as the prediction tools and database sequencing systems, the expression profiles and differentially expressed circRNAs have been quite different (Yan et al., 2018; Wang et al., 2019; Tang et al., 2020; Chen et al., 2021). With regard to single placental circRNAs in GDM pathophysiology, only hsa_circ_0005243 has been reported to be associated with trophoblast cell dysfunction. This circRNA is related to inflammation as well as to the proliferation and migration of trophoblast cells (Wang et al., 2020b). To date, the molecular mechanism of the circRNA-related etiology and pathophysiology of GDM, and especially the key circRNAs regulating autophagy in GDM placentas, remain largely unknown.

## Materials and methods

### Clinical specimens

The placentas in this study were from 66 maternal volunteers who underwent cesarean birth in the Renmin Hospital of Wuhan University from June 2021 to March 2022. Six maternal volunteers were selected for high-throughput sequencing and differentially expressed circRNA screening [3 normal control (NC) and 3 GDM cases], and the samples from the remaining 60 samples (30 NC and 30 GDM cases) were used for verification. Pregnant women with multiple pregnancies, preterm delivery, infection, chronic liver and kidney diseases, thyroid diseases, family history of diabetes and other endocrine diseases were excluded. The diagnosis of GDM was conducted by oral glucose tolerance test (75 g) in the second trimester of pregnancy (24–28 weeks of pregnancy). All volunteers gave birth to full-term infants (gestational age 38–41 weeks). Fresh placentas obtained from the mothers were washed quickly and gently with PBS several times, immediately frozen in liquid nitrogen and preserved. The clinical characteristics of the enrolled pregnant women are listed in Tables 1, 2. The mean age was 30.25 ± 3.04 years old, ranging from 25 to 36 (*n* = 66). Each participant gave informed consent. This study was approved and supervised by the ethics committee of the Renmin Hospital of Wuhan University. The ethics license number is WDRY2022-K118. All experiments were conducted in accordance with the Code of Ethics of the World Medical Association.

**TABLE 1:** Clinical data for the GDM patients and NCs for sequencing.

	GDM (*n* = 3)	NC (*n* = 3)	*p* value
Maternal age (years)	31.00 ± 3.00	32.33 ± 1.53	0.53
Gestational age (weeks)	38.77 ± 0.74	39.2 ± 0.52	0.452
Prepregnancy BMI	21.03 ± 1.36	23.81 ± 0.59	0.031
BMI at delivery	25.21 ± 1.49	28.96 ± 1.93	0.056
SBP, mmHg	105.67 ± 2.52	121.33 ± 7.64	0.028
DBP, mmHg	64.00 ± 7.21	77.33 ± 6.35	0.075
HBA1c (%)	5.43 ± 0.45	4.77 ± 0.25	0.089
Hemoglobin (g/L)	128.00 ± 7.00	125.67 ± 16.77	0.835
ALT (U/L)	9.33 ± 1.53	7.00 ± 1.73	0.155
AST (U/L)	16 ± 2.65	12.67 ± 1.53	0.132
TBA (µmol/L)	1.73 ± 0.39	2.64 ± 0.76	0.138
FBG (mmol/L)	4.51 ± 0.60	4.27 ± 0.48	0.609
1-h OGTT (mmol/L)	10.63 ± 0.54	6.24 ± 1.58	0.01
2-h OGTT (mmol/L)	8.98 ± 1.73	5.31 ± 0.46	0.024

**TABLE 2:** Clinical data for the GDM patients and NCs in the validation cohort.

	GDM (*n* = 30)	NC (*n* = 30)	*p* value
Maternal age (years)	30.47 ± 3.23	30.03 ± 2.87	0.585
Gestational age (weeks)	38.95 ± 0.61	38.97 ± 0.63	0.885
Prepregnancy BMI	21.71 ± 1.63	22.79 ± 1.67	0.014
BMI at delivery	25.27 ± 1.71	28.11 ± 1.29	<0.0001
Gestational weight gain (kg)	9.17 ± 1.70	13.02 ± 3.14	<0.0001
SBP, mmHg	115.6 ± 8.43	124.8 ± 6.27	<0.0001
DBP, mmHg	70.20 ± 8.19	73.5 ± 7.71	0.114
HBA1c%	5.51 ± 0.26	4.91 ± 0.23	<0.0001
Hemoglobin (g/L)	113.73 ± 8.97	111.97 ± 7.98	0.424
ALT (U/L)	10.43 ± 2.42	9.23 ± 1.61	0.027
AST (U/L)	15.53 ± 2.18	13.97 ± 2.37	0.01
TBA (µmol/L)	2.45 ± 0.87	2.58 ± 0.52	0.512
FBG (mmol/L)	4.84 ± 0.39	4.39 ± 0.36	<0.0001
1-h OGTT (mmol/L)	10.16 ± 1.12	7.69 ± 0.95	<0.0001
2-h OGTT (mmol/L)	8.29 ± 0.96	6.58 ± 0.77	<0.0001
Neonatal birth weight (g)	3483.17 ± 295.41	3396.33 ± 239.86	0.216

### RNA sequencing

Total RNA was isolated from 3 NC to 3 GDM cases using TRIzol reagent (Thermo Fisher Scientific, 15596026) and then treated with DNase I following the manufacturer’s instructions. RNA integrity was determined with a Qubit3.0 and a Qubit™ RNA Broad Range Assay kit (Life Technologies, Q10210). Two micrograms of total RNA was used for stranded RNA sequencing library preparation using a Ribo-off rRNA Depletion Kit (Illumina, MRZG12324) and a KC-Digital™ Stranded mRNA Library Prep Kit for Illumina^®^ (Seqhealth Co., Ltd., DR08502) following the manufacturer’s instructions. The kit eliminates duplication bias in the PCR and sequencing steps by using a unique molecular identifier (UMI) of 8 random bases to label the preamplified cDNA molecules. The library products corresponding to 200–500 bp were enriched, quantified and finally sequenced on a NovaSeq 6000 sequencer (Illumina) with the PE150 model.

### Identification and quantification of placental circular RNAs

The sequences were mapped to the reference genome of human from the Ensembl browser (http://ftp.ensembl.org/pub/current_fasta/homo_sapiens/dna/Homo_sapiens.GRCh38.dna.toplevel.fa.gz) using STAR software (version 2.5.3a) with the default parameters. The unmapped reads were used to predict circRNAs with find_circ (version 1.2) and CIRCexplorer software (version 2.3.0). The intersecting results predicted by these two software programs were the final circRNAs. The expression of circRNAs was quantified with in-house scripts, and then the spliced reads per billion mapped (SRPBM) values were calculated. CircRNAs differentially expressed between groups were identified using the edgeR package (version 3.12.1). A FDR cutoff of 0.05 and a fold-change cutoff of 2 were used to judge the statistical significance of circRNA expression differences (Supplementary Tables S3, S4).

### Functional annotation, target microRNA and interaction network prediction

The target miRNAs and mRNAs of the differentially expressed circRNAs were predicted by miRanda (version v3.3a). Then, the ceRNA network was constructed by igraph (version 1.1.2). Gene Ontology (GO) and Kyoto Encyclopedia of Genes and Genomes (KEGG) enrichment analyses of targeted mRNAs were implemented with KOBAS software (version: 2.1.1) with a corrected *p* value cutoff of 0.05 to judge statistically significant enrichment (Supplementary Tables S5, S6).

### Quantitative real-time polymerase chain reaction analysis

Total RNA was extracted from placental tissues using the TRIzol method and was reverse-transcribed using a HiScript II 1st Strand cDNA Synthesis Kit (+gDNA Wiper) (Vazyme, China) according to the manufacturer’s protocol. The cDNA synthesis reaction conditions were as follows: 50°C for 15 min and 85°C for 2 min.

Then, we randomly selected 12 differentially expressed circRNAs (fold change ≥ 2, FDR < 0.05) from the RNA sequencing results for validation, including 6 upregulated and 6 downregulated circRNAs. The primers were designed and synthesized by Tsingke Biotechnology Co., Ltd. (Tsingke, China). Their sequences are listed in Supplementary Table S1. Real-time polymerase chain reaction (qRT–PCR) was performed using ChamQ Universal SYBR qPCR Master Mix (Vazyme, China) on a Quantagene q225. GAPDH was used as a housekeeping gene. The reaction was performed in a total volume of 10 μl containing 1 μl of cDNA, 10 μM of forward and reverse primers and 5 μl of 2× ChamQ Universal SYBR qPCR Master Mix. The reaction conditions were as follows: 95°C for 30 s followed by 40 cycles at 95°C for 10 s and 60°C for 30 s. The relative quantity of circRNA expression was calculated using the 2^−ΔΔC^_T_ method. The experiments were repeated in triplicate. Finally, the PCR products were verified by agarose gel electrophoresis and purified for Sanger sequencing. The details of primers used in this study are presented in the Supplementary Table S1.

### RNase R treatment

Total RNA was digested with RNase for 15 min at 37°C and immediately transferred to ice. The control group RNA was digested with ddH_2_O at the same time and temperature. Then, qRT–PCR was performed as described above. The relative expression of circRNAs and mRNAs was calculated using the 2_T_^−ΔΔC^ method.

### PolyA^+^ RNA extraction

PolyA^+^ RNA was enriched using a Magnosphere™ UltraPure mRNA Purification Kit (Takara, China) according to the manufacturer’s protocol. Then, polyA^+^ RNA fragments were immediately transferred to 1.5 ml Ep tubes and stored at −80°C until experiments were carried out.

### Cell culture and transfection

Cell lines were purchased from the Committee of the Type Culture Collection of the Chinese Academy of Sciences. HTR-8/SVneo cells were cultured in RPMI-1640 medium (HyClone, United States) supplemented with 10% FBS (v/v, Gibco, United States), 100 IU/ml penicillin and 100 μg/ml streptomycin (Gibco, United States) at 37°C in a humidified atmosphere with 5% CO_2_. In the HG treatment experiment of HTR-8/SVneo cells, D-glucose was dissolved in the supplemented medium up to the indicated final glucose concentration. HTR-8/SVneo cells were transiently transfected using Lipofectamine 3000 (Invitrogen, United States). The siRNA interference sequence was designed and synthesized by Geneseed Biology Co., Ltd. (Guangzhou, China). The specificity of the siRNA was detected by qRT–PCR. An siRNA control was used as a negative control. The siRNA sequences are shown in Supplementary Table S1.

### Immunofluorescence

Immunofluorescence staining was performed on vertical paraffin sections using ULK1 and LC3II antibodies. Briefly, placental tissues were fixed in 4% paraformaldehyde for 24 h. The samples were dehydrated for paraffin embedding, and 3 μm sections were cut. The vertical placental sections were dewaxed in xylene, rehydrated and subjected to antigen retrieval in a microwave oven with 0.01 M sodium citrate buffer (pH = 6.0). The sections were blocked with goat serum for 1 h at room temperature. Then, the sections were incubated overnight in a humidified chamber at 4°C with primary antibodies and washed with PBS three times. The sections were subsequently incubated with the secondary antibodies for 1 h at room temperature. DAPI was used to counterstain the nuclei. Images were obtained using a FluoView 1000 microscope (Olympus, Japan). The details of antibodies used in this study are presented in the Supplementary Table S2.

### Western blotting

The placental tissues or cells were lysed using cell lysis buffer for Western and IP (Beyotime, China) supplemented with protease inhibitor (cOmplete™, EDTA-free Protease Inhibitor Cocktail, Merck, Germany). Protein was quantified using a BCA kit (Beyotime Biotechnology, China) according to the manufacturer’s protocol. Forty micrograms of protein were separated by 10% SDS-polyacrylamide gel electrophoresis (SDS–PAGE). After blocking for 1 h, the membranes were incubated with primary antibodies at 4°C in a tabletop incubator. Then, the membranes were washed with PBS three times before being incubated with the secondary antibodies for 1 h at room temperature. Enhanced chemiluminescence (ECL) solution (Clarity™ Western ECL Substrate, Bio-Rad) was used to visualize the bands, and the bands were analyzed with the ImageJ software package (http://imagej.nih.gov/ij/). The details of antibodies used in this study are presented in the Supplementary Table S2.

### Statistical analysis

All data are described as the mean ± standard deviation (SD). Statistical analyses were performed using one-way ANOVA or *t* test using SPSS 12.0 software. *p* < 0.05 was considered to indicate statistical significance.

## Results

### General characteristics of circular RNA in human placental tissues

**FIGURE 1:**
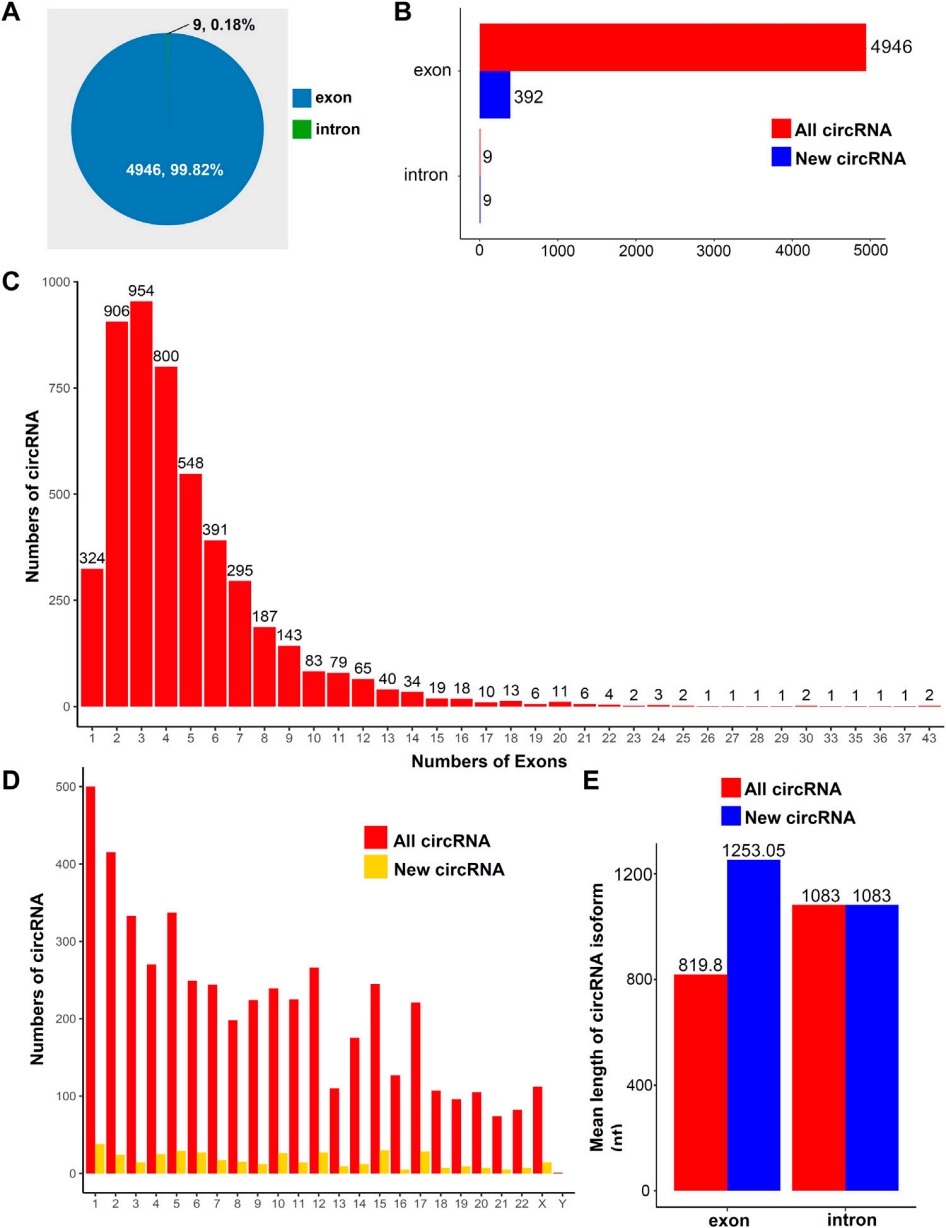
Overview of circRNA expression in human placental tissues. **(A)** Distributions of different types of circRNAs. **(B)** Distributions of new circRNAs or all circRNAs. **(C)** Distributions of exons in all circRNAs. **(D)** Distributions of circRNA on chromosomes. **(E)** Average lengths of all and new circRNAs.

To study the general characteristics of all circRNAs in the human placentas, we preliminarily analyzed the sequencing data. A total of 4955 circRNAs were identified in placental tissues. Most circRNAs were exonic circRNAs (99.82%, Figure 1A); only a small fraction of circRNAs were intronic (0.18%, Figure 1A). The distributions of new circRNAs and all circRNAs are presented in Figure 1B. Among all exonic circRNAs, there were up to 43 exons in two circRNAs, whereas most exonic circRNAs comprised 2–4 exons (Figure 1C). Based on the distributions of circRNAs on chromosomes, we found that both known circRNAs and new circRNAs were widely distributed across all chromosomes. The chromosome with the most distributed RNAs was chromosome 1 (Figure 1D). The mean lengths of exonic circRNAs among all circRNAs and new circRNAs were 819.8 and 1253.05 nt, respectively (Figure 1E). The mean length of intronic circRNAs among all circRNAs and new circRNAs was 1083 nt (Figure 1E). The details for all the circRNAs identified in this study are provided in Supplementary Table S3.

### Analysis of differentially expressed circular RNA

**FIGURE 2:**
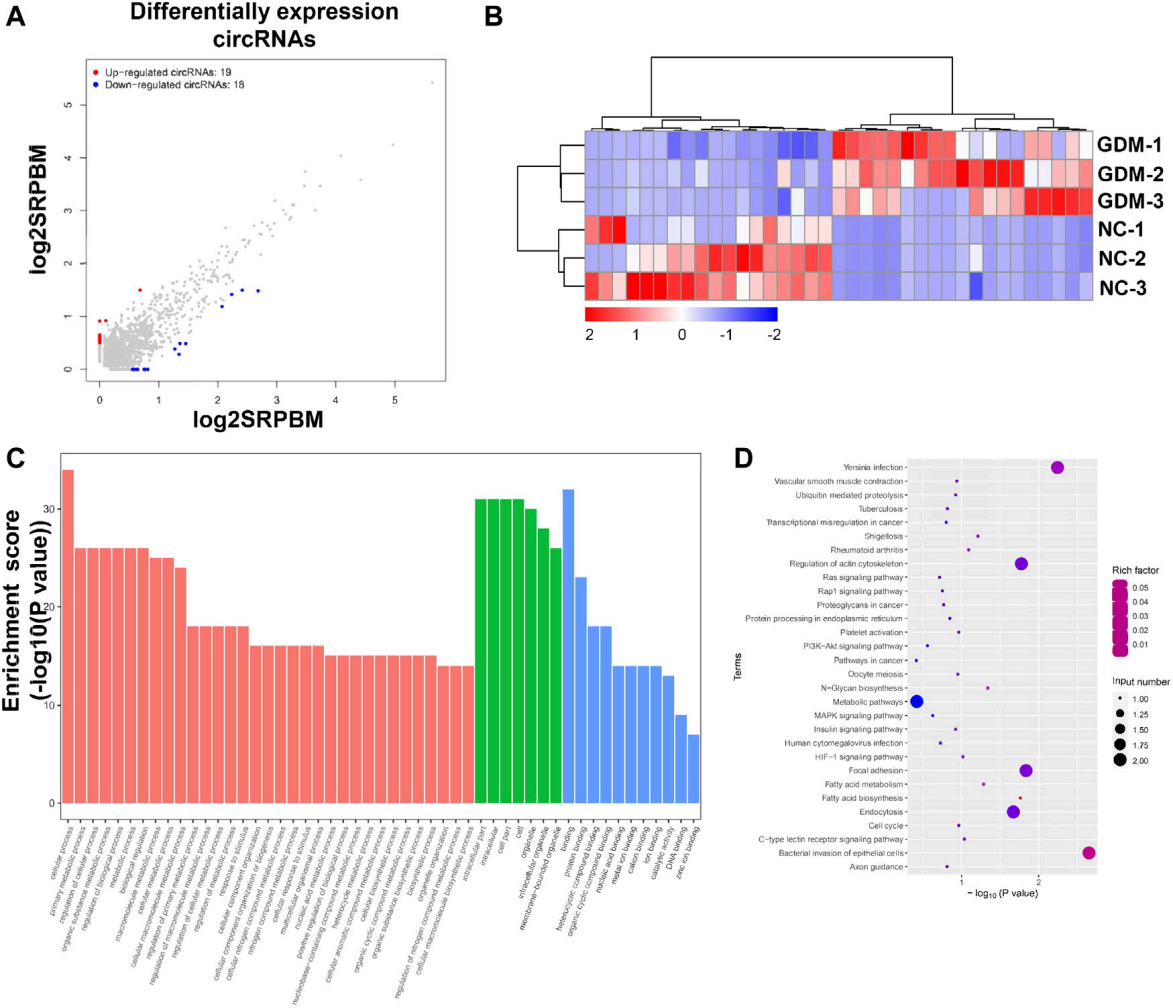
Annotation of differentially expressed circRNAs and GO and KEGG pathways of host genes. **(A)** Scatter plot showing the deregulated circRNAs in NC and GDM placentas. Significantly regulated genes had an FDR ≤ 0.05 and a fold change ≥ 2. **(B)** Heatmap showing the differences in circRNA expression between NC and GDM placentas. **(C)** Top 50 GO terms for parent genes with significantly differentially expressed circRNAs. **(D)** KEGG pathway enrichment analysis of parent genes with significantly differentially expressed circRNAs. The top 20 KEGG signaling pathways are annotated.

To understand the impacts and molecular changes of GDM placentas, we analyzed differentially expressed circRNAs through RNA sequencing. We found that 37 circRNAs were deregulated in GDM placentas compared with NC placentas (FDR < 0.05, fold change ≥ 2), among which 19 circRNAs were upregulated and 18 circRNAs were downregulated (Figures 2A,B; Supplementary Table S4). To explore the biological functions of these significantly differentially expressed circRNAs, GO term enrichment was performed. The top 50 significantly enriched biological process, cellular component, and molecular function terms included cellular process, primary metabolic process, regulation of cellular process, organic substance metabolic process, intracellular part, intracellular, cell part, cell and binding, and protein binding (Figure 2C; Supplementary Table S5). Further KEGG pathway enrichment analysis revealed that the deregulated circRNAs were enriched in pathways related to the PI3k-Akt signaling pathway, the insulin signaling pathway and the metabolic pathway (Figure 2D; Supplementary Table S5).

### Validation of differentially expressed circular RNA

**FIGURE 3:**
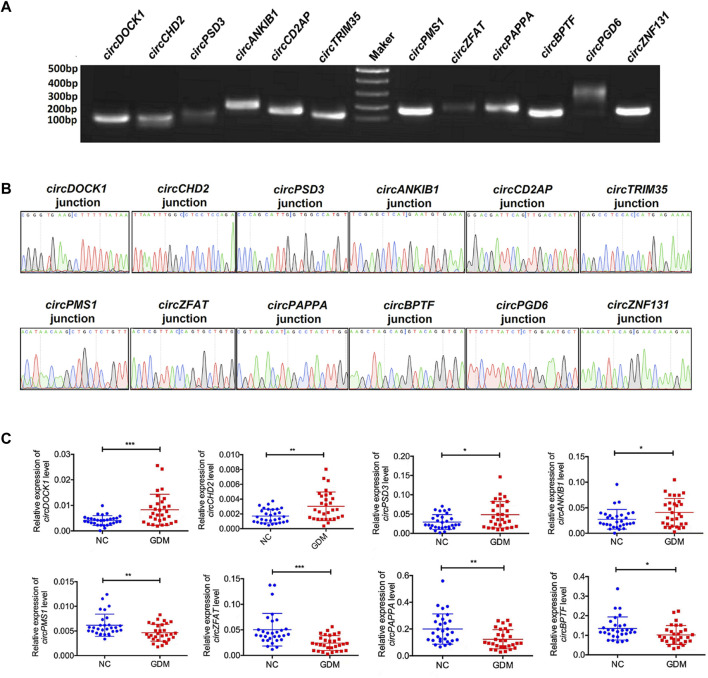
Validation of the results of RNA sequencing. **(A)** PCR products were contirmed by 2% agarose electrophoresis. **(B)** Sanger sequencing was used to confirm the circRNA primers. **(C)** qRT-PCR validation of 8 differentially expressed circRNAs in 60 female volunteers. *p value < 0.05, **p < 0.01, ***p < 0.001.

To validate the sequencing results, we next selected 12 differentially expressed circRNAs, including 6 upregulated circRNAs, *circDOCK1* (Chr10:126970701–127061776), *circCHD2* (Chr15:93000511–93009323), circPSD3 (Chr8:18799294–18804898), *circANKIB1* (Chr7:92294888–92327900), *circCD2AP* (Chr6:47503279–47554766), and *circTRIM35* (Chr8:27294079–27294310) and 6 downregulated circRNAs, *circPMS1* (Chr2:189791789–189818180), *circZFAT* (Chr 8:134600435–134610655), *circPAPPA* (Chr9:116207455–116235637), *circBPTF* (Chr17:67945408–67975958), *circPGD6* (Chr12:95208842–95211267), and *circZNF131* (Chr5:43161248–43161931), for further confirmation. Both agarose gel electrophoresis and Sanger sequencing methods were used to confirm the specificity of the PCR primers (Figures 3A,B). Indeed, qRT–PCR analyses showed that the expression patterns of these 12 differentially expressed circRNAs were consistent with the sequencing results. These results revealed that the expression levels of these circRNAs were significantly deregulated in GDM placentas compared with controls (Figure 3C).

### General biological properties of placenta-derived circular RNA

**FIGURE 4:**
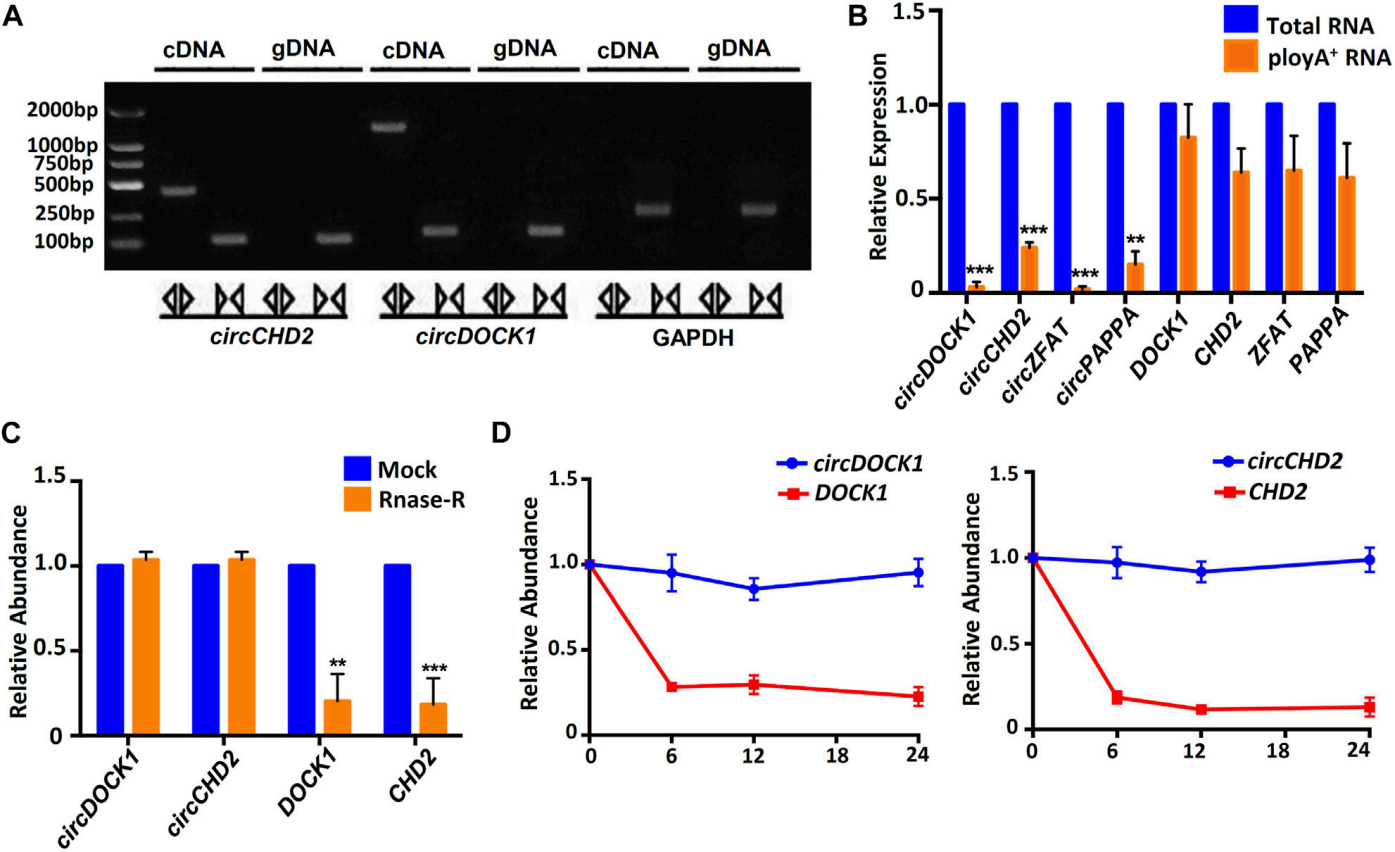
General biological properties of placenta-derived circRNAs. **(A)** Agarose gel electrophoresis of circRNA and linear RNA amplification products. **(B)** Total RNA-derived cDNA revealed successful amplification of circRNA, while the polyA^+^ RNA-derived cDNA did not. Four circRNAs were examined, and the corresponding linear transcripts were used as controls. **(C)** Analysis of circRNA resistance to RNase R digestion. Two circRNAs were examined, and the two corresponding linear RNAs were used as controls. **(D)** Human placental tissue-derived circRNAs were stably expressed within 24 h, while the corresponding linear transcripts degraded rapidly.

Unlike linear RNA, circRNA is mainly formed by reverse splicing at the 3′ and 5′ ends, so circRNA has unique biological characteristics. We amplified circRNA and linear RNA from cDNA and gDNA using different primers. The results showed that linear RNA could be amplified from cDNA or gDNA, but circRNA could be amplified only from cDNA. GAPDH was used for the control. The PCR products were validated on a 2% agarose gel (Figure 4A). Four circRNAs, *circDOCK1*, *circCHD2*, *circZFAT* and *circPAPPA*, and their linear RNAs were amplified by total RNA and polyA^+^ RNA-derived cDNA. The results showed that both total RNA and polyA^+^ RNA could be used to amplify the linear RNAs but not the circRNAs, indicating that the circRNAs had a unique circular structure (Figure 4B).

Then, we selected two circRNAs, *circDOCK1* and *circCHD2*, and two corresponding linear RNAs, *DOCK1* and *CHD2*, for control studies. The qRT–PCR results showed that after 15 min of RNase digestion, the expression levels of all linear RNAs were significantly decreased, while those of circRNAs remained unchanged (Figure 4C). This indicated that the circRNAs were resistant to RNase R digestion, while the linear RNAs were sensitive to RNase R digestion.

To test the stability of the circRNAs, we next detected two circRNAs, *circDOCK1* and *circCHD2*, and their corresponding linear RNAs, *DOCK1* and *CHD2*, at different time points within 24 h. Both linear RNAs showed significant degradation from 6 h. The circRNAs exhibited good stability within 24 h (Figure 4D). These results indicated that the circRNAs were more stable than the linear RNAs.

### Hyperglycemia promotes autophagy in the placentas of gestational diabetes mellitus patients

**FIGURE 5:**
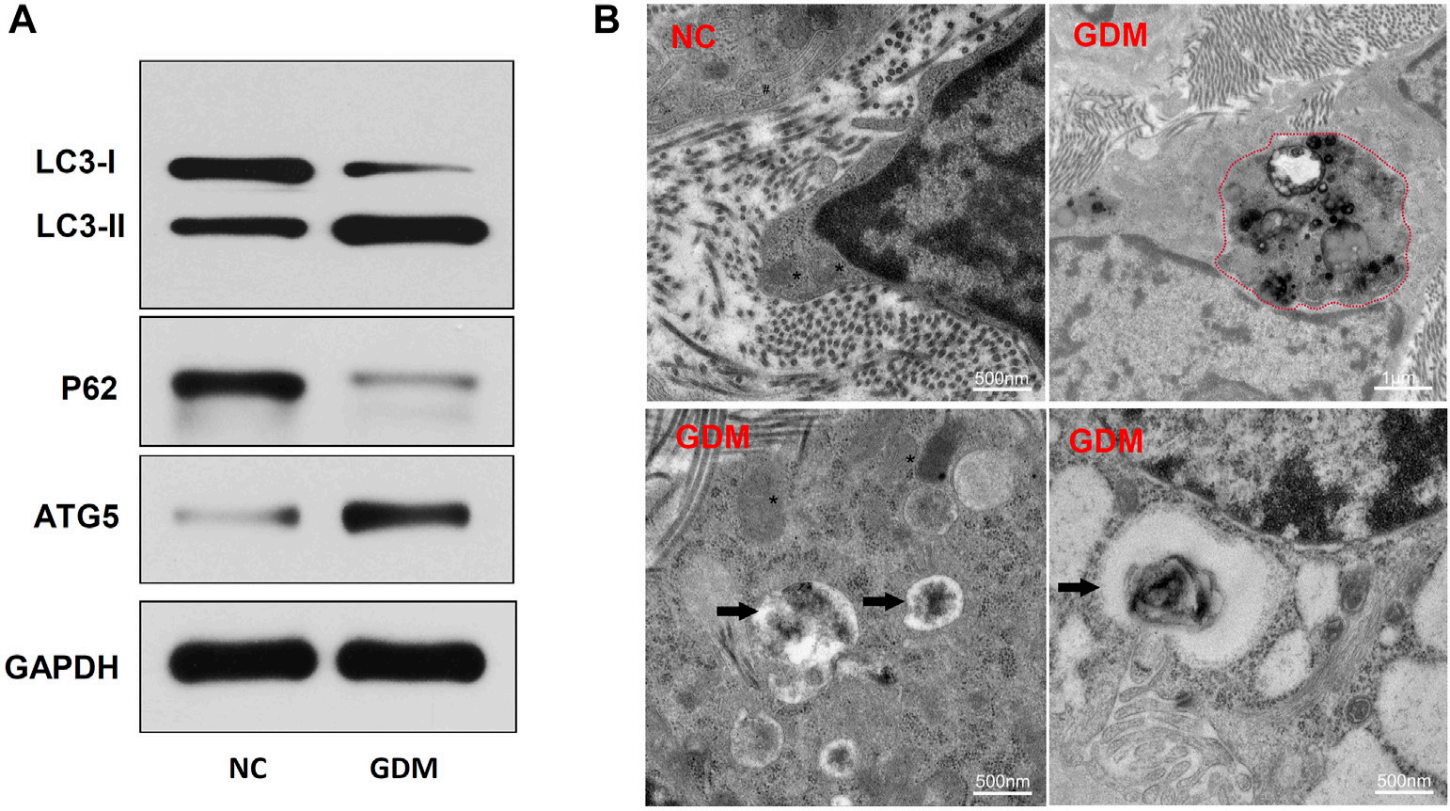
Placenta autophagy-related protein expression and ultrastructure in NCs and patients with GDM. **(A)** The expression of the autophagy-related proteins LC3-I, LC3-II, p62, and ATG5 was determined by western blotting. **(B)** TEM of NC and GDM placental tissues. Integrated and clear organelles, such as mitochondria (asterisks) and the endoplasmic reticulum (number signs), were present, but autophagosomes were not seen in NC samples. The GDM placentas showed obvious autolysosome structures (black arrowheads), and partially degraded mitochndria were visible in the autophagolysosomes (red dotted line).

To identify autophagy levels in the placentas of patients with GDM, we first detected the expression levels of autophagy-related proteins. The results showed that LC3-II and ATG5 were significantly upregulated and that p62 was downregulated in the GDM group compared with the control group (Figure 5A). LC3-II is a marker of autophagy activation, and ATG5 is involved in autophagy formation. These results suggested that placental autophagy was enhanced in the GDM patients. To further confirm the autophagy activation in the placentas of GDM patients, transmission electron microscopy (TEM) analysis was performed. TEM revealed that placentas from GDM patients showed obvious autophagy-associated structures, such as autolysosomes (Figure 5B). These results suggest the enhancement of autophagy in GDM placentas compared to NC placentas.

### Prediction of the autophagy-associated circCDH2-miRNA-mRNA interaction axis

**FIGURE 6:**
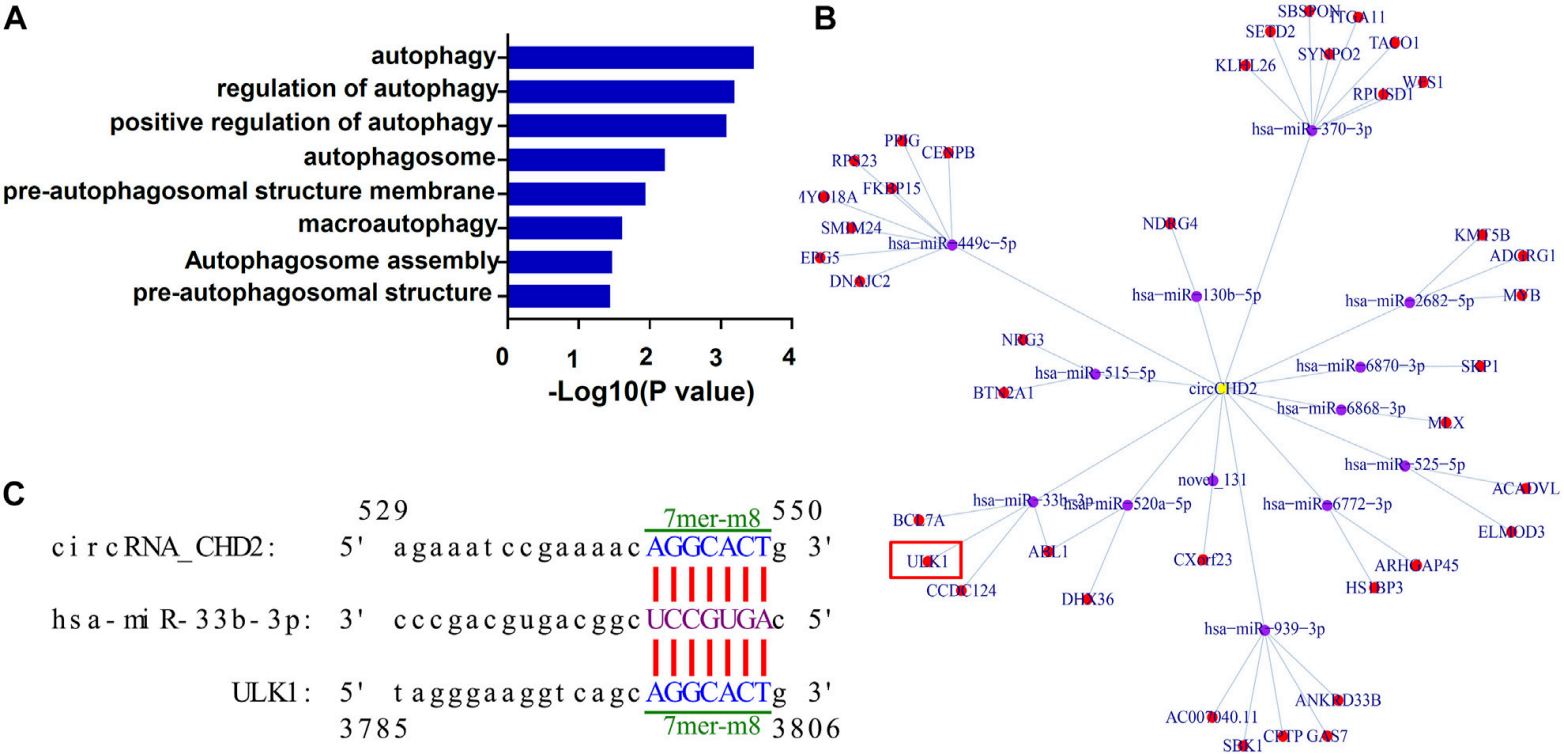
Prediction of the autophagy-associated circCDH2-miRNA-mRNA interaction network. **(A)** GO analysis of the autophagy-related terms of circRNA traget genes. **(B)** A circCDH2-miRNA-mRNA interaction network was predicted using bioinformatics online programs (starBase, circBase, TargetScan, and miRBase). Yellow indicates circCDH2, purple indicates miRNA, and red indicates the target gene. **(C)** The predicted binding sites between circCDH2-miR-33b-3p and miR-33b-3p-ULK1 are shown. The 7mer-m8 was an exact match to positions 2–8 of the mature miRNA (seed + position 8), and the 8mer was an exact match to positions 2–8 of the mature miRNA (seed + position 8) followed by a “G”.

CircRNAs located in the cytoplasm is one of competitive endogenous RNA (ceRNA) and act as miRNA sponge, thus regulating expression of the corresponding target gene (Chen, 2016). To explore circRNAs that may be involved in autophagy regulation, we next performed GO enrichment analysis on the target genes of differentially expressed circRNAs. The results showed that many of the target genes were enriched for autophagy-related terms, such as “autophagosome assembly” and “pre-autophagosomal structure membrane” (Figure 6A; Supplementary Table S6). Through analysis of the prediction results, 12 autophagy related differentially expressed circRNAs were used to construct the circRNA-miRNA-mRNA regulatory network (Supplementary Table S7). According to the results of the bioinformatics prediction, upregulated *circCDH2* may function as a ceRNA to suppress the inhibitory effects of miR-33b-3p on ULK1, thus resulting in its upregulated expression. This results suggesting that the *circCDH2–miR-33b-3p–ULK1* interaction axis may be associated with autophagy in GDM placentas (Figure 6B). The predicted binding sites between circCDH2–miR-33b-3p and miR-33b-3p–ULK1 are shown in Figure 6C.

### The *circCDH2-miR-33b-3p-ULK1* interaction axis may be associated with autophagy in the placentas of gestational diabetes mellitus patients

**FIGURE 7:**
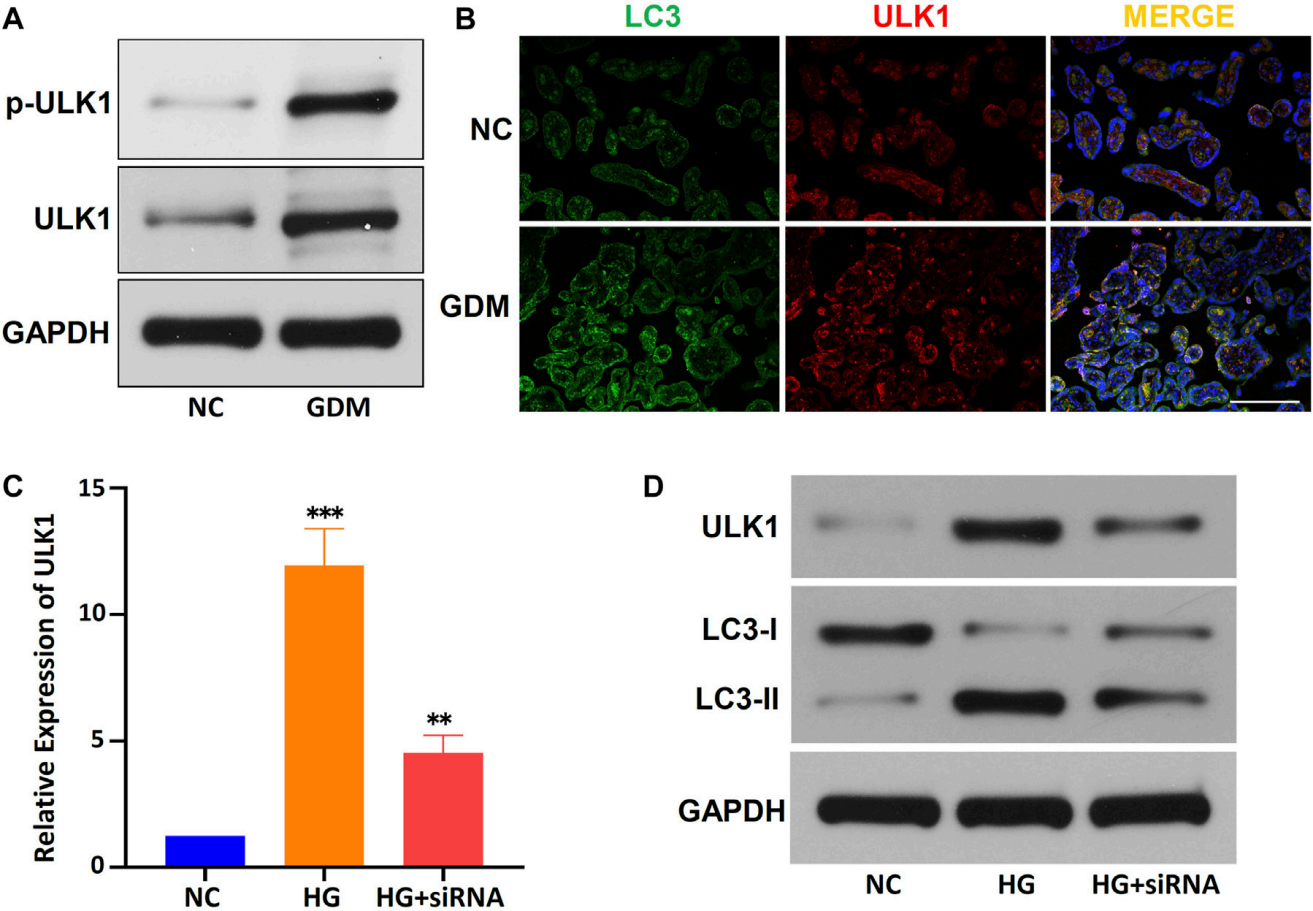
The circCDH2-miR-33b-3p-ULK1 interaction axis may be associated with autophagy. **(A)** The protein expression and phosphorylation (S555) of ULK1 were determined using western blotting. **(B)** Co-immunofluorescence staining for ULK1 (red) and LC3 (green) in placental sections from control and GDM patients is shown. Nuclei were stained with DAPI. Scale bars=100 μm. **(C)** The relative expression of ULK1 mRNA in HG-treated HTR-8/SVneo cells and the inhibitory effects of siRNAs on circCDH2 expression were examined were examined by qPCR. **(D)** Western blot analysis showed the expression of LC3 and ULK1 in cells treated with HG.

Furthermore, the average levels of the p-ULK1 (Ser555) and ULK1 proteins were significantly higher in the placentas of GDM patients than in the placentas of NCs, the average expression level of *miR-33b-3p* was down-regulated in GDM placentas (Figure 7A; Supplementary Table S8). Immunofluorescence showed that ULK1 was colocalized with LC3 (a marker of autophagy) in GDM patients, and the expression of ULK1 was upregulated in GDM tissues (Figure 7B). To further verify the role of ULK1 in the regulation of GDM placental autophagy, we used a HG-treated HTR-8/SVneo cell line and *circCDH2* siRNA *in vitro*. The relative expression of *ULK1* in HG-treated HTR-8/SVneo cells was examined by qPCR, and the results showed that ULK1 was significantly upregulated after HG treatment and that siRNA significantly reduced the expression of *ULK1*, expression level of miR-33b-3p corresponds to ULK1 expression (Figure 7C; Supplementary Table S8). Then, the expression of ULK1 protein and LC3 protein was determined in HTR-8/SVneo cells cultivated in HG medium. ULK1 and LC3-II were significantly upregulated in HG-treated HTR-8/SVneo cells, and their expression in HTR-8/SVneo cells was reduced after downregulation of circCDH2 (Figure 7D). These results indicate that the *circCDH2–miR-33b-3p–ULK1* interaction axis may be associated with autophagy.

## Discussion

GDM is a leading disease in pregnant women whose prevalence is increasing worldwide (Choudhury and Devi Rajeswari, 2021). Although several studies have shown that autophagy is necessary for placental development, the levels and functions of autophagy in GDM remain controversial (Martino et al., 2016; Avagliano et al., 2017; Ji et al., 2017; Wang et al., 2020a; Han et al., 2020; Hung et al., 2020; Ji et al., 2020; Diceglie et al., 2021). In addition, the roles of circRNAs, which are novel ncRNAs that participate in a variety of pathological processes by acting as miRNA sponges, in the pathogenesis of gestational diabetes are largely unknown. In this study, 4,955 human placenta-derived circRNAs were identified and annotated using RNA-seq analysis. Further bioinformatics analyses showed that deregulated circRNAs were mainly related to various metabolic processes as well as intracellular parts and binding in GDM placental tissues. We also found aberrantly enhanced autophagy levels in patients with GDM and the HTR-8/SVneo cell line exposed to excess glucose. Through GO enrichment analysis and circRNA interference, we found that the *circCDH2–miR-33b-3p–ULK1* interaction axis may be associated with autophagy activation in the placentas of GDM patients.

A few recent studies have reported that circRNAs may play important roles in the placenta in GDM (Yan et al., 2018; Wang et al., 2019; Tang et al., 2020). Consistent with these studies, we also found 37 differentially expressed circRNAs in GDM placentas. The circRNAs identified in this study were mainly exonic circRNAs (99.82%), and most of them were novel circRNAs, indicating that these circRNAs may be specifically expressed in the placental tissues of GDM patients. Unlike linear RNAs, circRNAs have a closed loop structure and no polyA^+^ tail and are highly insensitive to ribonuclease. Our results showed that the circRNAs were more resistant to RNase digestion than linear RNAs, which confirms these characteristics of circRNAs. However, including our findings, the expression profiles of total and differentially expressed circRNAs in related studies have differed some extent. These differences may be due to the different analysis tools, regions, populations and database sorting systems. In general, our study suggests that circRNAs may play important roles in the occurrence of GDM.

The possible biological functions of circRNAs were further elucidated by bioinformatics analysis. Through GO and KEGG pathway annotation, we found that multiple enrichment pathways were closely related to metabolic processes as well as islet signaling pathways. It is well known that the insulin signaling pathway is involved in the regulation of glucose homeostasis (Filippi et al., 2013). Moreover, insulin resistance is considered to be the main cause of GDM (Eckel et al., 2005; Song et al., 2013). These results suggest that circRNAs may affect the occurrence of GDM by regulating the insulin pathway, but the specific mechanism remains to be clarified.

Under physiological conditions, autophagy can regulate energy metabolism, including glucose metabolism and lipid metabolism (Kim and Lee, 2014). A series of metabolic changes occur in pregnant women, such as significant increases in the levels of hormones, glucose and free fatty acids in the blood, to accommodate the physiological changes during pregnancy and meet the needs of fetal growth and development (Liang et al., 2020). Under normal circumstances, B cells or EVTs can resist these internal environmental changes and maintain normal physiological functions through autophagy (Nakashima et al., 2013; Sarparanta et al., 2017). However, the development of autophagy in GDM placentas is still unclear. Our findings indicated that HG may increase autophagy in GDM, and the expression of LC3-II and ATG5 was significantly increased, while that of p62 was decreased, in GDM placentas (Figures 5A,B). Similarly, autophagy was activated in HG-treated HTR-8/SVneo cells (Figure 7D). Previous studies have shown that hyperglycemia causes ROS accumulation in trophoblasts of placental tissue, which may lead to excessive autophagy and aggravate insulin resistance. Autophagic vesicles in the cytoplasm of placental villous trophoblasts increase in number; inhibit villous microvessel formation; and lead to placental villus edema, structural damage and reductions in numbers, leading to the occurrence of GDM (He et al., 2016; Ji et al., 2017). Our study supports the idea that placental autophagy is increased in GDM patients.

Autophagy is related to tissue homeostasis, cellular metabolism and human disease and is regulated by multiple mechanisms, such as the ULK1 pathway and the Beclin1 pathway. The ULK1 complex plays an important role in the initiation of autophagy, by phosphorylation of ULK1, ULK1 complex is activated (or inhibited, dependent on specific phosphorylation sites) at critical times in response to upstream signals. Following activation, the ULK1 complex recruits downstream Atg proteins and Beclin-1 to autophagosome-forming sites, and manages autophagosome formation. In the subsequent elongation and maturation, LC3 and ATG12 ubiquitin-like conjugation systems are involved (Mizushima et al., 2008; Singh and Cuervo, 2011; Wirth et al., 2013). The molecular details of this process are being revealed, but the precise regulatory mechanism of ULK1 is currently unknown. Through high-throughput sequencing and bioinformatics prediction, we found that *circCDH2* may bind to a downstream miRNA by acting as a molecular sponge and further regulating ULK1 (Figure 6). Further analysis and verification showed that the expression of ULK1 was significantly upregulated in GDM placentas and HG-treated HTR-8/SVneo cells, and the Ser555 phosphorylation of ULK1 was also increased in GDM placentas (Figure 7). Thus, we speculate that *circCDH2* may be related to ULK1-mediated effects in GDM. However, the underlying mechanisms of the *circCDH2–miR-33b-3p–ULK1* interaction axis need further analysis.

Taken together, our findings constitute evidence that circRNAs may be involved in the pathology of the placenta in GDM patients compared with normal pregnant women. However, the diverse biological functions of circRNAs in the placenta need further exploration. We also found that circCDH2 may participate in the regulatory network of autophagy activation by regulating the expression of ULK1 in GDM patients and HG-treated HTR-8/SVneo cells. To a certain extent, our research expands the existing knowledge of GDM and provides new insights into the pathogenic mechanisms of GDM.

## Data Availability

Total RNA sequencing data are deposited in the NCBI Sequence Read Archive (SRA) database (GSE206041): https://www.ncbi.nlm.nih.gov/geo/query/acc.cgi?acc=GSE206041.
