# Supplementary material for: Identification of human placenta-derived circular RNAs and autophagy related circRNA-miRNA-mRNA regulatory network in gestational diabetes mellitus
Source: Front Genet. 2022 Nov 30;13:1050906. doi: 10.3389/fgene.2022.1050906 (PMC9748685; doi:10.3389/fgene.2022.1050906)
Supplement: Supplementary file 2 [file Table1.DOCX]

| Target | Sequence(5'-3) | Application |
| --- | --- | --- |
| circDOCK1 | F: TGTATGAAAACAAGGGAGAGGCT | RT-qPCR |
|  | R: CTGGCATCATAGTTATAAAAAGCTTC |  |
| circCHD2 | F: ATCAAACTCTGGAGGAGGCCA | RT-qPCR |
|  | R: CTTCTGATGGATTATCTGAATGCCT |  |
| circPSD3 | F: CCGATCTACATGGCCACCAAT | RT-qPCR |
|  | R: CCTTTTGGCTGCTTCCACATT |  |
| circANKIB1 | F: TTAAGGCCTCAGGATCTTCG | RT-qPCR |
|  | R: TGGTGGTTGTATTTCCCATGT |  |
| circCD2AP | F: TTGGGACTGTTTCCCTCAAAT | RT-qPCR |
|  | R: CCTGATGATTTCTCCAACTCG |  |
| circTRIM35 | F: GACGTTTCTTTTCTCATGGTGG | RT-qPCR |
|  | R: AGTTGCTTCTGCCTTGTCTCCT |  |
| circPMS1 | F: AAAAAGATCCAAGATCTCCTCA | RT-qPCR |
|  | R: CACTGACCACCGAAGTGATG |  |
| circZFAT | F: TGGCCTCCTTTCCTGAGA | RT-qPCR |
|  | R: CCACCGATGGCTGTGACT |  |
| circPAPPA | F: GCCCCTGAAGTATAAGGTGGT | RT-qPCR |
|  | R: TGCTACTCCTGCCAACTCCT |  |
| circBPTF | F: AAGGCTTCAAAGCTAGCAGGTAC | RT-qPCR |
|  | R: CTGGACCCACACTTGATGACTG |  |
| circPGD6 | F: GAGGTGGGGCTGGCTTAT | RT-qPCR |
|  | R: AGCATGGAGGACGCTGAT |  |
| circZNF131 | F: CATGGAAACAGCACCTAAATTG | RT-qPCR |
|  | R: GGGAGCTGAGTTTTCTTTGTTC |  |
| GAPDH | ACAACTTTGGTATCGTGGAAGG | RT-qPCR |
|  | GCCATCACGCCACAGTTTC |  |
| circDOCK1- divergent | F: GCACATCTTAGAAACATATGAAGGGT | PCR |
|  | R: CATGATGTTGAAGAGGGCATC |  |
| circDOCK1- convergent | F: GATGCCCTCTTCAACATCATG | PCR |
|  | R: ACCCTTCATATGTTTCTAAGATGTGC |  |
| circCHD2- divergent | F: GAAAACAAAGTGCCCAGGCTG | PCR |
|  | R: TCCGATTTTCAGCAGGCAGTTC |  |
| circCHD2- convergent | F: GAACTGCCTGCTGAAAATCGGA | PCR |
|  | R: CAGCCTGGGCACTTTGTTTTC |  |
| GAPDH- divergent | F: GAAGGTGAAGGTCGGAGTC | PCR |
|  | R: GAAGATGGTGATGGGATTTC |  |
| GAPDH- convergent | F: GCTGAGTACGTCGTGGAGTC | PCR |
|  | R: GAGAACAGTGAGCGCCTAGTG |  |
| DOCK1 | F: ACCGAGGTTACACGTTACGAA | RT-qPCR |
|  | R: TCGGAGTGTCGTGGTGACTT |  |
| CHD2 | F: AGCCAACCTTAACTGAGGAGT | RT-qPCR |
|  | R: GGCAAGTTGATTGGAGGGATG |  |
| ZFAT | F: CTCTTCTCACCAAATCAGTCGG | RT-qPCR |
|  | R: GGGTTCAGGTGTACTCAGAGG |  |
| PAPPA | F: GGATGGGTCATGGGCATTCA | RT-qPCR |
|  | R: GAAAAAGTAGCGTGGATCTCTGT |  |
| ULK1 | F: AGCACGATTTGGAGGTCGC | RT-qPCR |
|  | R: GCCACGATGTTTTCATGTTTCA |  |
| circCHD2-  siRNA | Sense: GAGAUCCUGUGAGAAGGGAAUUUGA | circCHD2  knockdown |
|  | Antisense: UCAAAUUCCCUUCUCACAGGAUCUC |  |
| siRNA-  NC | Sense: GAGGUCCGAGUGGAAUAAGUUAUGA | circCHD2  knockdown |
|  | Antisense: UCAUAACUUAUUCCACUCGGACCUC |  |
