# Supplementary material for: Identification of human placenta-derived circular RNAs and autophagy related circRNA-miRNA-mRNA regulatory network in gestational diabetes mellitus
Source: Front Genet. 2022 Nov 30;13:1050906. doi: 10.3389/fgene.2022.1050906 (PMC9748685; doi:10.3389/fgene.2022.1050906)
Supplement: Supplementary file 3 [file Table8.docx]

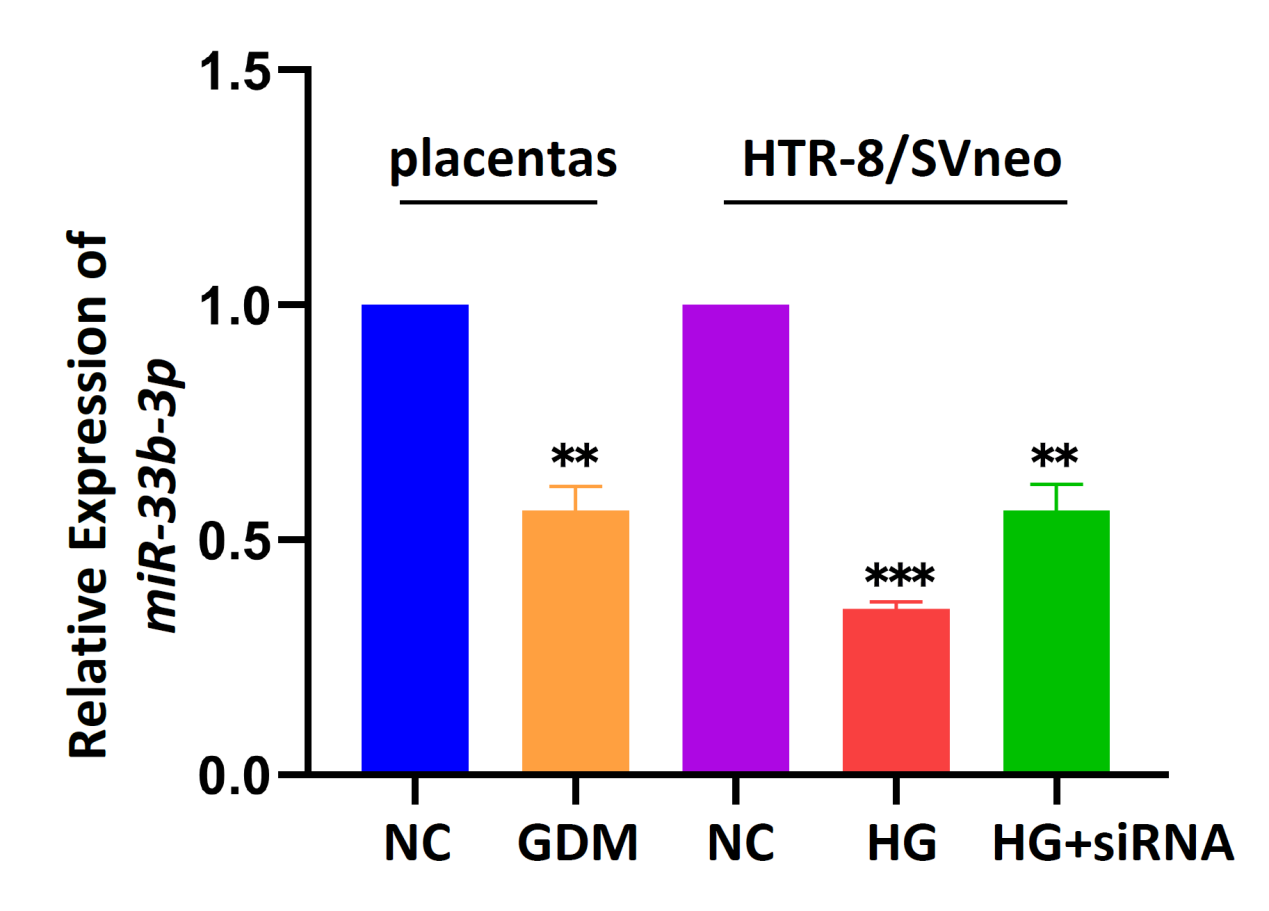
**Supplementary Table S8** The relative expression of miR-33b-3p in GDM placentas and HG-treated HTR-8/SVneo cell lines and the inhibitory effects of siRNAs on circCDH2 expression were examined by qPCR.
