# Supplementary material for: Identification of human placenta-derived circular RNAs and autophagy related circRNA-miRNA-mRNA regulatory network in gestational diabetes mellitus
Source: Front Genet. 2022 Nov 30;13:1050906. doi: 10.3389/fgene.2022.1050906 (PMC9748685; doi:10.3389/fgene.2022.1050906)
Supplement: Supplementary file 5 [file Table7.docx]

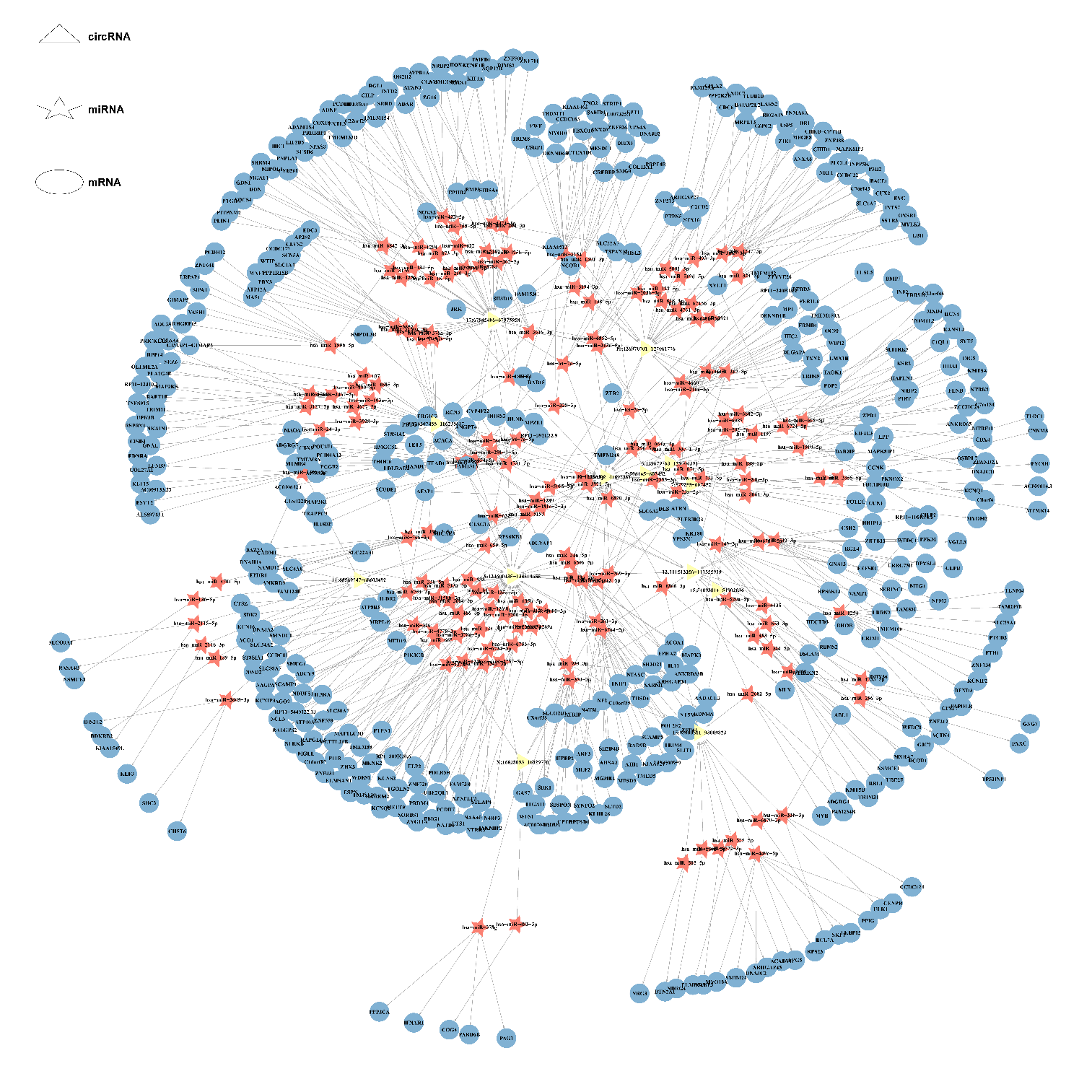


**Supplementary Table S7** The circRNA-miRNA-mRNA regulatory network of 12 autophagy related circRNAs in placentas. The yellow triangle, red pentagram and blue circle represent circRNAs, mRNAs, and miRNAs respectively. circRNA - circular RNA; miRNA - microRNA; mRNA - messenger RNA.
