# Supplementary material for: Identification of human placenta-derived circular RNAs and autophagy related circRNA-miRNA-mRNA regulatory network in gestational diabetes mellitus
Source: Front Genet. 2022 Nov 30;13:1050906. doi: 10.3389/fgene.2022.1050906 (PMC9748685; doi:10.3389/fgene.2022.1050906)
Supplement: Supplementary file 8 [file Table2.DOCX]

| antibody | source | manufacturer | Cat. Number | dilution rate |
| --- | --- | --- | --- | --- |
| LC3 (IF) | Mouse | abcam | ab244210 | 1:100 |
| ULK1 (IF) | Rabbit | Abclonal | A8529 | 1:100 |
| ULK1(WB) | Rabbit | CST | #8054 | 1:2000 |
| Phospho-ULK1 (Ser555) | Rabbit | CST | #5869 | 1:1000 |
| ATG5 | Rabbit | CST | #12994 | 1:2000 |
| P62 | Rabbit | abcam | ab109012 | 1:3000 |
| LC3(WB) | Rabbit | CST | #4108 | 1:1000 |
| GAPDH | Rabbit | abcam | ab181602 | 1:10000 |
| Cy3–conjugated Affinipure Goat Anti-Rabbit IgG(H+L) | Goat | Proteintech | SA00009-2 | 1:100 |
| CoraLite488-conjugated Goat Anti-Mouse IgG(H+L) | Goat | Proteintech | SA00013-1 | 1:100 |
| HRP-Goat anti Rabbit | Goat | ASPEN | AS1107 | 1:10000 |
| HRP-Goat anti Mouse | Goat | ASPEN | AS1106 | 1:10000 |
